# Bullying at Workplace and Brain-Imaging Correlates

**DOI:** 10.3390/jcm7080200

**Published:** 2018-08-04

**Authors:** Giovanni Nolfe, Mario Cirillo, Alessandro Iavarone, Alberto Negro, Elisabetta Garofalo, Annisa Cotena, Massimo Lazazzara, Gemma Zontini, Sossio Cirillo

**Affiliations:** 1 Department of Mental Health Naples 1, Work Psychopathology Medical Centre, Naples 89122, Italy; annisa.cotena@libero.it (A.C.); massi956@alice.it (M.L.); gemmazontini@gmail.com (G.Z.); 2 Neuroradiology Service, Department of Medical, Surgical, Neurological, Metabolic & Aging Sciences, University of Campania “Luigi Vanvitelli”, Naples 80131, Italy; emmecirillo@libero.it (M.C.); alberto.negro@hotmail.it (A.N.); 3 Neurological and Stroke Unit, Centro Traumatologico Ortopedico Hospital, AORN “Ospedali dei Colli”, Naples 80131, Italy; aleiavarone@gmail.com (A.I.); elisa.garofalo@libero.it (E.G.); 4 Department of Precision Medicine, University of Campania Luigi Vanvitelli, Naples 80131, Italy; cirillos@alice.it

**Keywords:** adjustment disorders, bullying at workplace, neuroimaging, hippocampus

## Abstract

The relationship between psychosocial stress at work and mental health outcome is well-known. Brain-imaging studies hypothesize morphological brain modifications connected to work-related stress. To our knowledge this is the first study describing the link between work characteristics and brain imaging in a sample of work-related psychiatric patients assessed according to standardized clinical and diagnostic criteria. The aims of the study are: (1) to evaluate hippocampal and whole brain volumes in work-related psychiatric disturbances; (2) to verify the relationship between brain changes and the anxious and/or depressive symptoms; (3) to observe the relationship between the brain changes and the degree of the bullying at workplace. The hippocampus and whole brain volumes of 23 patients with work-related adjustment-disorders were compared with 15 controls by means of MRI. MR images highlight a smaller hippocampal volume in patients compared with controls. Significant reduction in the patients’ gray matter was found in three brain areas: right inferior temporal gyrus, left cuneus, left inferior occipital gyrus. The reduction of the hippocampi volumes was related to work distress and, above all, to bullying at workplace. The results confirm that the morphological brain abnormalities could be involved in work-related psychiatric disturbances.

## 1. Introduction

The relationship between work stress and psychiatric disturbances is a well known datum [1,2]. Many studies [3,4,5,6] have observed the development of depressive, anxiety and adjustment disorders related to negative working environment: organizational dysfunctions of work (stress, overload, effort-reward imbalance, high job demands with low decision latitude and low social support) as well as interpersonal work-related psychological distress (bullying at workplace, mobbing). The Kungsholmen Project [7] has observed, in a population-based follow up study, that the high job stress experiences were related to higher risk of dementia and Alzheimer disease in late life (independent of other known risk factors). The increased risk of dementia and Alzheimer has been confirmed by Andel et al. [8]. Therefore, it’s possible to hypothesize that work-related stressors could activate the process of brain aging and that the negative working conditions could have adverse effects on cognitive functioning and mental health overall.

However, the underlying pathophysiological mechanisms involved are still unclear. About this topic, although the researches focused on the role of the hypothalamic-pituitary-adrenal (HPA) axis function have produced inconclusive results [9], some findings suggest that workplace-bullied individuals have a reduced tonic hypothalamic pituitary axis (HPA) activity [10,11] and that the chronic development of the work-related distress leads to hypocortisolism similarly to individuals living under other chronic stress conditions and to patients with post-traumatic stress disorder (PTSD) or with stress-related bodily disorders [12].

Many data suggest that the limbic system (amygdala, anterior prefrontal cortex, hippocampus) regulates the HPA activity and that the human response to acute stress is mediated by these structures [13,14]; furthermore, the chronic psychosocial stress induces the inhibition of hippocampal neurogenesis and the development of depressive symptoms [15,16]. The results of a recent positron emission tomography (PET) study [17] are in agreement with these observations as they have found a reduction in the 5-HT1_A_ receptor binding in some limbic structures (hippocampus, anterior cingulate cortex and anterior insular cortex). Blix et al. [9] have observed, by means of magnetic resonance imaging (MRI), a significant reduction of the anterior cingulate cortex as well as the dorsolateral prefrontal cortex and, furthermore, the volume reduction (related to the degree of work-related stress) in the caudate and in the putamen. Also, workers suffering from occupational stress showed altered functional couplings within the emotion- and stress-processing limbic networks [18].

Further support to these issues is given by studies demonstrating relevant brain changes in other stress-related pathologies [19,20,21,22] and by evidences from several MRI studies which reported decreased hippocampal volume in major depressive disorder [23], that is the psychiatric illness strongly linked to negative work environment [6].

The hypothesis that work-related stress and bullying at workplace could induce structural brain changes has then a significant, although not conclusive, theoretical basis.

According to these clinical and theoretical perspectives, aims of our study are: (1) to evaluate the hippocampal structure and volume in subjects with well-defined work-related psychiatric disturbances (adjustment disorders); (2) to verify if the morphological brain changes are produced by the work stress and bullying at workplace “per se” or if they are related to the anxious and/or depressive psychopathological degree of the clinical feature; (3) to describe the relationship between the brain structural changes and the degree of the organizational stress at work and/or of the bullying at workplace strength.

## 2. Subjects and Methods

The study was carried out on 23 patients who approached the Work Psychopathology Medical Centre of the Department of Mental Health of Naples (Italy) between 2014 and 2015. They were 12 man and 11 women, with a mean age 50.96 years (SD = 9.93) and a mean education 13.74 years (SD = 3.11). Each subject was included in a clinical trial in order to carry out the diagnoses and to evaluate the pathogenic degree of bullying at work and each patient have provided informed consent. According to Leyman [24] bullying at workplace consists of hostile acts, oppressions, psychological and/or physical harassment, and repeated and persistent attacks on work and personal identity carried out against the individual to exclude him or her from the working group.

The 15 control subjects (NC) were eight men and seven women, mean age = 51.67 years (SD = 9.82) and mean education = 14.14 years (SD = 3.18). All NC were mentally healthy as assessed by the Structure Clinical Interview-axis I SCID-I non-patient edition [25] and comparable to patients according to occupational levels. 

Exclusion criteria were: previous significant neurological and/or psychiatric disorders (dementia, stroke, epilepsy, Parkinson’s disease, depressive or bipolar disorder, psychosis etc.), alcohol or substances abuse or drugs significantly interfering with mood and/or cognition.

All control subjects gave written informed consent to MRI scanning and received generic information about the study, since they were blind of the precise goal of it.

The psychiatric diagnoses of patients were carried out by means of semi-structured clinical interviews according to the Diagnostic and Statistical Manual of mental disorders-4th-text revised (DSM IV-TR) diagnostic criteria. The anxious and depressive dimensions of the clinical features were evaluated by means of the Hamilton Anxiety Scale (HAM-A) [26] and the Hamilton Scale for Depression (HAM-D) [27]. The life-event stressors were evaluated using the Holmes–Rahe Scale Social Readjustment Rating Scale (SRRS) [28]. Patients in which the non-work stressors score accounted for the at least 30% of the total SRRS score were not included in the final sample.

The subjective perception of distress and bullying at workplace was evaluated by the Naples Questionnaire of Work Distress (nQ-WD) [29]. This scale was validated in a sample of workers from Southern Italy, whose general and demographic characteristics were comparable to that of patients under investigation. The nQ-WD explores the main dimensions of work distress by means of three subscales. The subscale Harassment/Bullying at workplace (H) assesses dysfunctional phenomena more specifically tied to the negative interpersonal relationships; the Organizational Stress (OS) subscale evaluates features related to organizational anomalies on the whole; finally, the third subscale (BPS), gives a measure of the overall effects on the bio-psychosocial functioning of the worker.

### 2.1. Volumetric Analysis with Manual Segmentation of the Hippocampus

The manual segmentation of the hippocampal structures was carried out following the international harmonized protocol developed for the manual segmentation of the hippocampus on MR images by the centers of the European Alzheimer’s Disease Consortium (EADC) and the Alzheimer’s Association. This was performed using the interactive software MultiTracer developed at the Laboratory of Neuro-Imaging at the University of California, Los Angeles (Los Angeles, CA, USA). The manual segmentation of the hippocampus was used because it allows a better segmentation and partition analysis than automatic segmentation methods due to the anatomic complexity of the hippocampus, in which the gray and white substances are entwined through very narrow and tortuous connections.

The use of the European Alzheimer’s Disease Consortium (EADC) and Alzheimer’s Disease Neuroimaging Initiative (ADNI) harmonized protocol (HarP) EADC-ADNI HarP was defined by an international Delphi Panel for the manual segmentation of the hippocampus on MR. It is associated with high measurement stability versus traditional segmentation protocols, and good reproducibility within and among human tracers. Hippocampi segmented with the HarP can be used as reference for the qualification of human tracers and automated segmentation algorithms [30].

The results were placed in a table to be analyzed statistically with ANOVA.

### 2.2. Semiautomatic Volumetric Analysis with SIENAX

3D T1-weighted images of normalized volumes of the whole brain were obtained by means of a fully automated method called structural imaging evaluation of normalized atrophy methodology (Siena) [31], which allows measuring longitudinal and cross-sectional changes in brain volumes and is part of the Functional Magnetic Resonance Imaging of the Brain Software Library FMRIB (Functional Magnetic Resonance Imaging of the Brain) Software Library. In the current study, the cross-sectional version of this tool (SIENAX) was used. This software performs the separation of brain from non-brain tissue, estimates the outer skull surface and uses these results to drive the spatial transformation to a standard template for normalizing with regard to the skull size. Next, a probabilistic brain mask derived in standard space is applied to ensure that certain structures, such as eyes or optic nerves, have not been included in the brain segmentation. Finally, random field model based segmentation is used to further isolate different tissue types and to provide the normalized volumes of total brain, namely, grey matter (GM), white matter (WM) and cerebro-spinal fluid (CSF). Therefore, SIENAX allows us to obtain volumetric data about gray matter, white matter, cerebral spinal fluid, total cerebral and total normalized brain volume through the use of specfic command strings (the term “semiautomatic” underlines the functioning, partially operator-dependent, of this tool.

### 2.3. Automatic Volumetric Analysis with VBM

The Voxel Based Morphometry (VBM) Analysis was performed by means of the VBM8 toolbox of the Statistical Parametric Mapping software package with default parameters incorporating the DARTEL toolbox in order to obtain a high-dimensional normalization protocol [32]. Images were bias-corrected, tissue-classified, and registered by using linear (12-parameter affine) and nonlinear transformations (warping) within a unified model [32]. Subsequently, the warped GM segments were affine-transformed into Montreal Neurological Institute (MNI) space and were scaled by the Jacobian determinants of the deformations to account for the local compression and stretching that occurs as a consequence of the warping and affine transformation (modulated GM volumes) [33]. Finally, the modulated volumes were smoothed with a Gaussian kernel of 8-mm full width at half maximum. The GM volume maps were statistically analyzed by using the general linear model based on Gaussian random field theory. Statistical analysis consisted of an analysis of covariance (ANCOVA) with age and sex as covariates. The MNI152 (a.k.a., International Consortium for Brain Mapping—ICBM—152) atlas [32], incorporated into Statistical Parametric Mapping version number 8 (Functional Imaging Laboratory Wellcome Trust Centre for Neuroimaging Institute of Neurology, UCL 12 Queen Square, London WC1N 3BG, UK) SPM8, was used for localizing anatomically VBM results. Group differences in GM and WM volumes within the regions that were expected to show changes in stressed subjects, i.e., anterior cingulate cortex (ACC), medial prefrontal cortex (mPFC), hippocampus, amygdala and insular cortex were tested with VBM by restricting the search space to a mask encompassing the amygdalae (both sides), hippocampi, the ACC, the medial and superior frontal gyrus, and the insular cortex (both sides). This mask was derived using the WFU Pick Atlas (maldjian@wfubmc.edu), by adding the respective regional areas as defined by the atlas into a confluent, large mask.

### 2.4. Statistics

Descriptive statistics have been used to summarize data. The comparison between patients and NC in relation to measures of hippocampal volume (right and left) and whole brain (SIENAX) has been performed by one-way ANOVA, with significance level between hippocampal measures corrected by the number of comparisons on a Bonferroni basis. The statistical power (lambda) and size effect (Cohen’s d) have been checked. A normality test (Shapiro Wilk) has been applied on means of hippocampal measures of both patients and controls. The correlation between brain volumetric measures distinguishing patients from NC and measures assessing psychopathology and work-related distress have been performed by partial correlation matrix. Given the number of comparisons, only correlations at *p* = 0.01 have been considered significant.

## 3. Results

All patients were suffering from chronic Adjustment Disorders, namely, with depressed mood (7 cases), with anxiety (4 cases) and with mixed anxiety and depressed mood (12 cases). The average score on the HAM-A and HAM-D were, respectively, of 19.91 (SD 2.92) and 17.83 (SD 2.93).

The overall nQ-WD score was 54.98 (SD 8.69). The mean nQ-WD Subscales were: H = 24.93 (SD 6.67), OS = 12.74 (SD 4.57) and BPS = 17.43 (SD 3.22).

The manual segmentation of the hippocampal structures on MR images highlights the smaller volumes, of left as well as right hippocampus, in the group of patients as compared with normal controls. The right hippocampus volume of patients was 3441.5 (SD 3083.4) vs. the controls volume that was 5948.8 (SD 3193.9); the difference reached statistical difference (*F* = 5.731; *p* = 0.0222; lambda = 5.20; power = 0.60). The left hippocampus of patients also resulted smaller (3202.9, SD 2795.9) than that of controls (6137.5, SD 3079.9); this difference resulted highly significant (*F* = 9.052; *p* = 0.0048; lambda = 8.31; power = 0.82). The effect size for hippocampus was medium-large for the right (Cohen’s d = 0.78) and large for the left (Cohen’s d = 0.99). No significant difference between patients and controls was found on the whole brain volume (Brain Sienax). The results are shown in Table 1.

As somewhat expected, the Shapiro Wilk test showed a significant difference from normal distribution in patients both on right (*W* = 0.73; *p* < 0.0001) and left (*W* = 0.76; *p* < 0.0001) hippocampus measures, whereas in controls no significant difference was observed.

The partial correlation matrix showed slight inverse correlation between hippocampal volumes and overall nQ-WD scores (*r* = −0.467, *p* = 0.0237 for the right; *r* = −0.442, *p* = 0.0335 for the left hippocampus). However, this effect was almost exclusively due to the high inverse correlation of hippocampal volumes with the H subscale (*r* = −0.523, *p* = 0.0094 for the right; *r* = −0.527, *p* = 0.0088 for the left hippocampus), whereas no correlation approached significance with OS and BPS subscales, thus suggesting a possible relationship of hippocampal volume decrease with particular dimensions of work distress. 

Furthermore, no correlation was found between hippocampus volumes and the duration of work distress, nor with the level of depressive and anxious symptoms as measured by the HAM-A and HAM-D rating scales.

**Figure 1:**
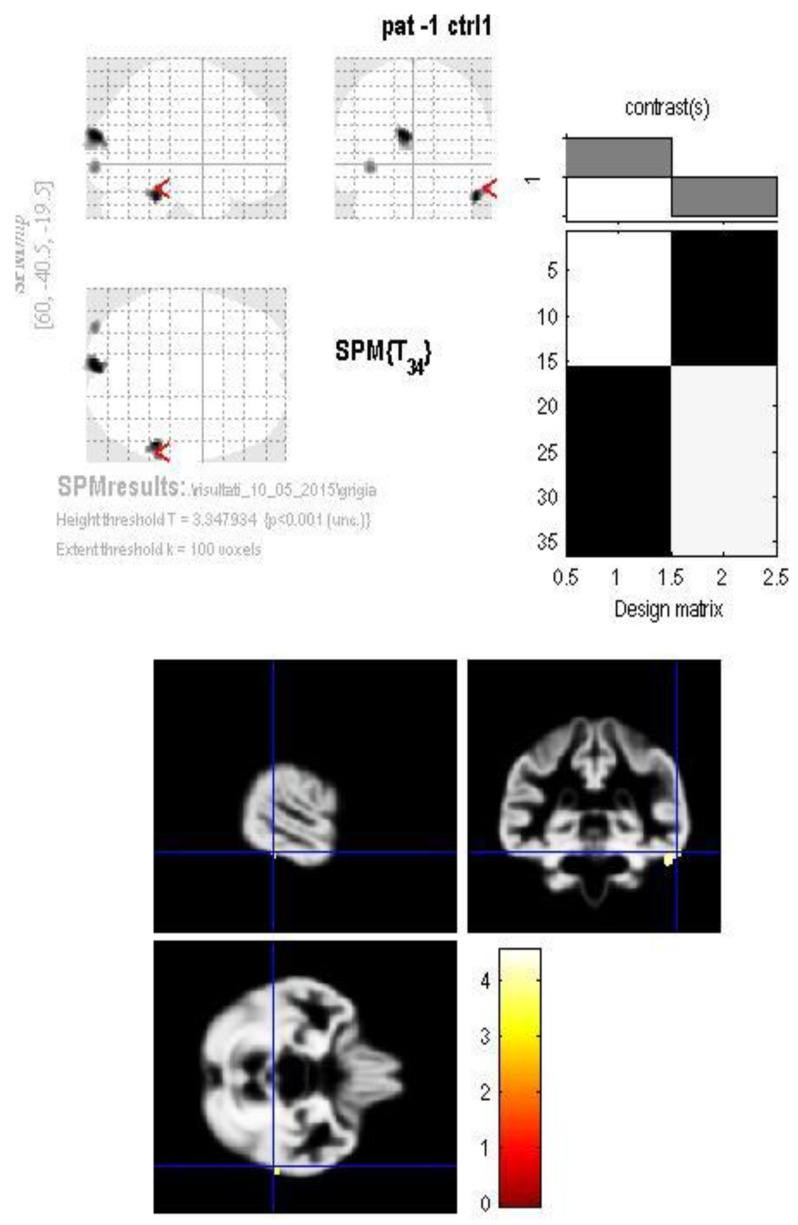
The GM volume group difference map shows a significant reduction in the right inferior temporal gyrus (Brodmann area 20) in the patients group compared to controls.

**Figure 2:**
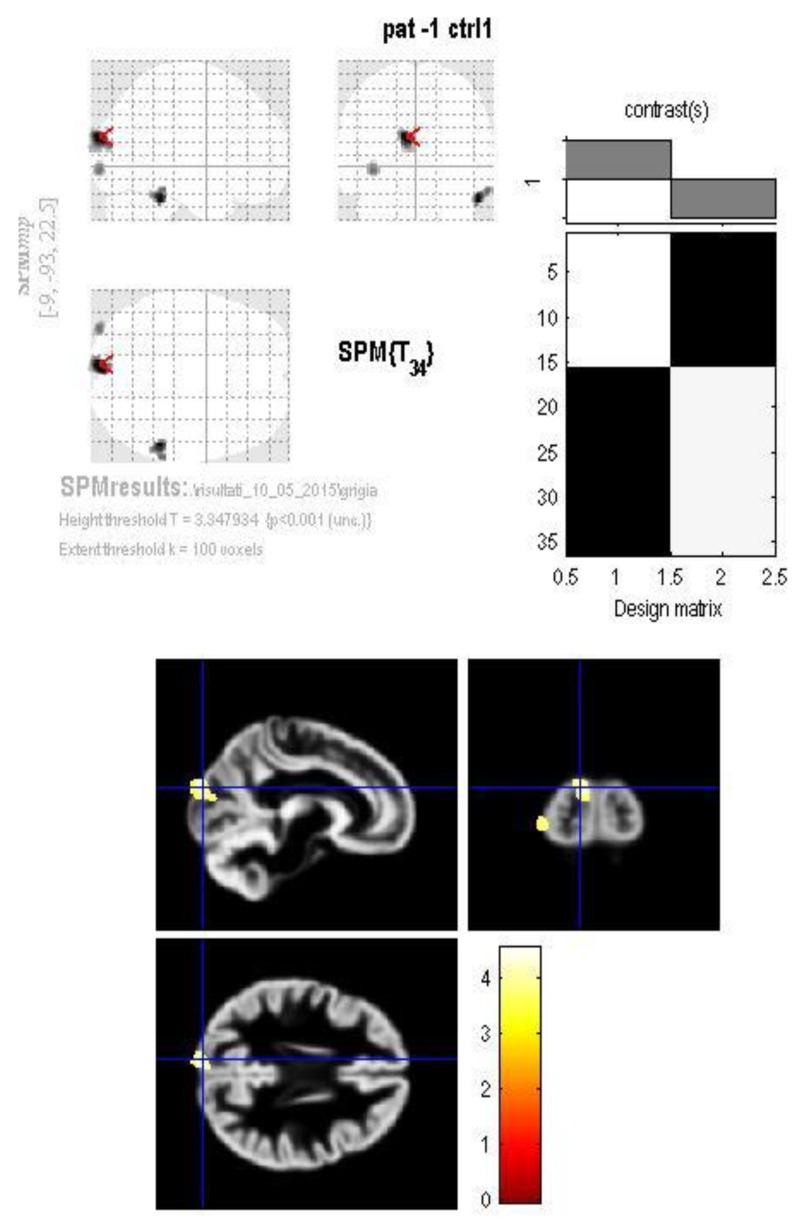
The GM volume group difference map shows a significant reduction in the left cuneus (Brodmann area 19) in the patients group compared to controls.

**Figure 3:**
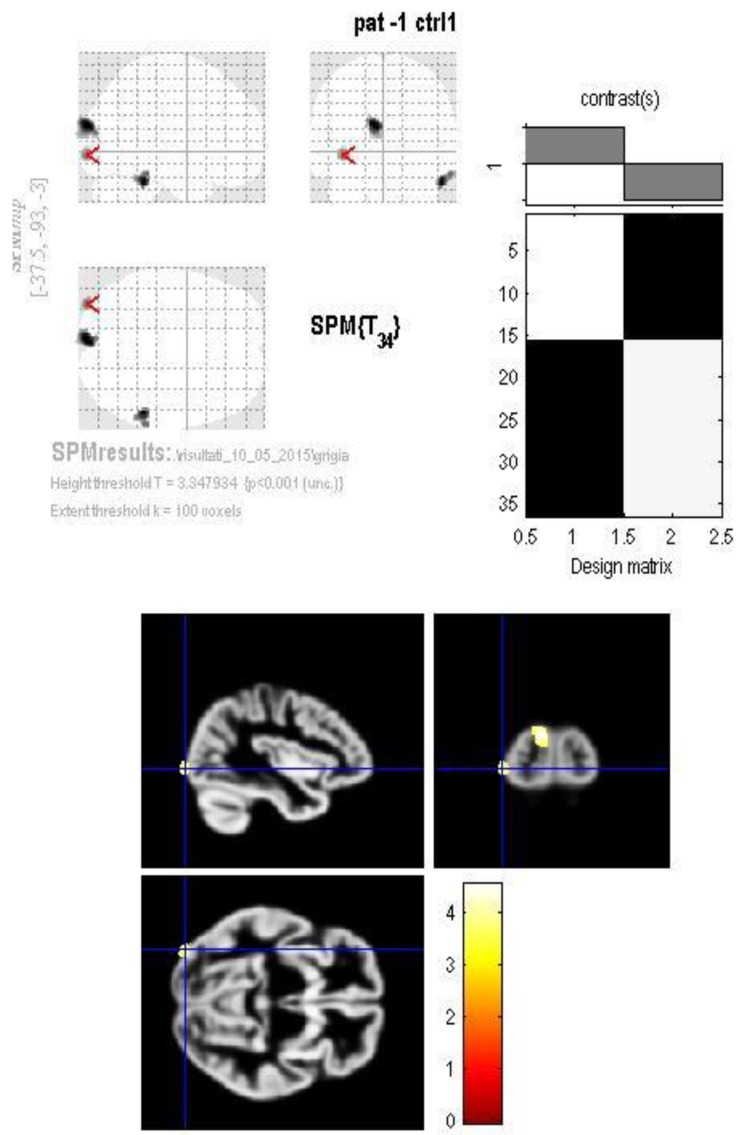
The GM volume group difference map shows a significant reduction in the left inferior occipital gyrus (Brodmann area 18) in the patients group compared to controls.

Significant reduction in the gray matter was found in three clusters among patients: in the right inferior temporal gyrus (Brodmann area 20) (Figure 1), in the left cuneus (Brodmann area 19) (Figure 2) and in the left inferior occipital gyrus (Brodmann area 18) (Figure 3).

## 4. Discussion

This report is, as far as we know, the first morphological study carried out in subjects suffering of working stress-related psychopathologies objectively evaluated by means standardized clinical and diagnostic criteria (structured clinical interviews according to the DSM IV diagnostic criteria) and by means of rating scales to measure the psychopathological dimensions of the psychiatric disorders (adjustment disorders). 

It confirms the relationship between working stress and the modification of the brain morphology, it underlines the significant volume reduction of the hippocampus, with a greater involvement of the left hemisphere. 

Our results are in agreement with the remark that the reduction of hippocampal volume is a consolidated marker of the stress-related psychopathologies as PTSD and depressive illness [21,34] and with the observation that the drugs used in these disorders also promote the neurogenesis in the hippocampus [35]. On the other hand, some researches observed the reduction of the grey matter in the anterior cingulate cortex and in the dorsolateral prefrontal cortex [9] as well as in the anterior cingulate, hippocampal and para-hippocampal gyrus [22]. Moreover, other studies showed that the hippocampal dysfunction in PTSD is lateralized with the left side being more impaired than the right [36].

Our data try to integrate, then, the heterogeneous and complex sequence of evidences that have underlined the involvement of the limbic structures in mediating between psychosocial stressors and their psychopathological effects and underline the potential role of the hippocampus related to the neurobiological effects of work-related psychosocial stress. Work stress, therefore, can be regarded as a life-event-stressor that, like other forms of acute and chronic stress, could induce changes of brain morphology.

A central topic of our research is the datum that shows that the reduction of hippocampal volume is not significantly correlated to the scores of scales that measure the severity of anxiety and depressive symptoms, but rather, to some characteristics of occupational stress. As is known, the psychiatric effects of work distress are frequently observed in the clinical practice and research and they are expressed above all through the psychopathological dimensions of anxiety and depression [3,4]. In the light of our results it can be hypothesized that hippocampal atrophy is related to occupational stress and to harassment at workplace, regardless of the role of the psychopathological dimensions of their effects.

Moreover, we observed a statistically significant link between the hippocampal atrophy and the working environment’s dysfunctional phenomena. This significant relationship is related to the work harassment and to anomalies of the interpersonal relationships (bullying at workplace) rather than to the phenomena more clearly related to organizational working stress. This datum highlights the hypothesis that the brain structure modifications could be more frequent in those psychiatric disorders linked to forms of occupational stress that act exclusively on the subject in its individuality than in cases related mainly to organizational dysfunction of the work as a whole. 

We found a slight difference between right and left hippocampus, being the left hippocampus more involved. This finding is in agreement with data from the literature highlighting a specific vulnerability of these structures to stress-related disorders [37]. Our data are unable to disentangle the question, which remains still unclear. However, we hypothesize that prevalence of verbally mediated inputs of distressing factors in our patients could play a role in overloading the left hemisphere, both at language and long-term memory processing levels.

As if the hippocampal reduction volume is concerned, this could hypothetically arise to a reduction of neurogenesis or to the effect of factors facilitating atrophic processes. The question of the real existence of neurogenesis in the hippocampus has been recently debated [38]. Also in this case, our data cannot add support to one position (decreased neurogenesis) or the other (promoting atrophy). In both cases, we would highlight the possible causal link between work distress and the detrimental effect on brain areas crucial for cognition and emotional processing.

Finally, we found a reduction in volume of the Brodmann areas 18, 19 and 20 that are connected to the dorsomedial and dorsolateral prefrontal cortex, towards the hippocampus, to the basal ganglia and to the amygdala. In effect, comparing by means the VBM analysis the morphological brain abnormalities in patients than in controls, we have found an involvement of these occipital-temporal areas that make up the ventral network of long-term memory known as “what pathway” [39]. We suggest that these changes could be associated with abnormalities of the semantic memory that mainly concerns the details of episodic autobiographical memory, as suggested in recent studies [40], about the life-events-stressors as working stress and, above all, as bullying at workplace. 

## 5. Limitations

(1) The rather small sample of the subjects investigated is a significant limitation of the study. Therefore, even though the sensitivity was enhanced by the use of a homogenous study group, is difficult to generalize the results.
(2) The cross-sectional method of this study makes difficult to demonstrate conclusively whether this brain characteristics are cause o consequence of work-related bullying.
(3) Patients did not undergo neuropsychological assessment since cognitive impairment was in the exclusion criteria; furthermore, although some patients referred subjective cognitive complaints, these were neither confirmed by informants nor supported by impaired performances at screening tools. However, we strongly believe that further studies could use neuropsychological evaluations to better substantiate the hypothesis of work-related abnormalities of the semantic memory. Follow-up studies are also needed to evaluate if the nature of these brain-imaging correlates is reversible or not.

## 6. Conclusions

There are limitations to the design of this explorative study but the results provide to suggest the possible association between work-related stress and brain changes.

The advantage of the present study is that it combines VBM analysis and investigations of structural volumes with a manual segmentation approach. In regions with poor white and gray matter demarcation, as in the hippocampus, the manual segmentation is regarded as more reliable than VBM and the two methods should, therefore, be used in tandem. Consequently, it is not surprising that manual segmentation showed reductions in the hippocampus while the corresponding GM and VM volumes did not differ from controls.

Work related-stress and bullying at workplace could be considered as a relevant life-event-stressors and could be associated to poor mental health outcomes in a wide group of workers. The new worldwide economic model of work, the ever increasing relevant role of job insecurity and injustice at workplace could be, currently, a significant variable about “global health”. We sustain with this study the need of achieving a wider knowledge of the pathogenetic mechanisms linked with these increasingly common disturbances of the contemporary societies.

## Figures and Tables

**Table 1:** Manual segmentation.

	Brain Sienax (Patients)	Brain Sienax (Controls)	Right Hippocampus (Patients)	Right Hippocampus (Controls)	Left Hippocampus (Patients)	Left Hippocampus (Controls)
	1,487,785.08 ± 70445.2	1,441,922.35 ± 75173.7	3441.5 ± 3193.9	5948.8 ± 3193.0	3202.9 ± 2795.9	6137.5 ± 3079.9
*f* value	3.582		5.731		9.052	
*p* value	*p* = 0.0667		*p* = 0.0222		*p* = 0.0048	
